# Mycobacteria Exploit Host GPR84 to Dampen Pro-Inflammatory Responses and Promote Infection in Macrophages

**DOI:** 10.3390/microorganisms13010110

**Published:** 2025-01-08

**Authors:** Reziya Wumaier, Ke Zhang, Jing Zhou, Zilu Wen, Zihan Chen, Geyang Luo, Hao Wang, Juliang Qin, Bing Du, Hua Ren, Yanzheng Song, Qian Gao, Bo Yan

**Affiliations:** 1 Key Laboratory of Medical Molecular Virology (MOE/NHC/CAMS), School of Basic Medical Sciences, Shanghai Medical College, Shanghai Public Health Clinical Center, Fudan University, Shanghai 200433, China; 16111010063@fudan.edu.cn; 2 Shanghai Institute of Infectious Disease and Biosecurity, Fudan University, Shanghai 200032, China; 23111010092@m.fudan.edu.cn; 3 Shanghai Public Health Clinical Center, Fudan University, Shanghai 201508, China; zhoujing@shaphc.org (J.Z.); wenzilu@shaphc.org (Z.W.); czhan2020@163.com (Z.C.); luogeyang@fudan.edu.cn (G.L.); wh94zz@163.com (H.W.); songyanzheng@shaphc.org (Y.S.); 4 Pathology Department, The First Affiliated Hospital of Shenzhen University, Shenzhen Second People’s Hospital, Shenzhen 518035, China; 5 Shanghai Frontiers Science Center of Genome Editing and Cell Therapy, Shanghai Key Laboratory of Regulatory Biology and School of Life Sciences, East China Normal University, Shanghai 200241, China; jlqin@bio.ecnu.edu.cn (J.Q.); bdu@bio.ecnu.edu.cn (B.D.); huaren@bio.ecnu.edu.cn (H.R.)

**Keywords:** tuberculosis (TB), G-protein-coupled receptors 84 (GPR84), pro-inflammatory cytokines

## Abstract

Tuberculosis (TB) remains the major cause of mortality and morbidity, causing approximately 1.3 million deaths annually. As a highly successful pathogen, *Mycobacterium tuberculosis* (*Mtb*) has evolved numerous strategies to evade host immune responses, making it essential to understand the interactions between *Mtb* and host cells. G-protein-coupled receptor 84 (GPR84), a member of the G-protein-coupled receptor family, contributes to the regulation of pro-inflammatory reactions and the migration of innate immune cells, such as macrophages. Its role in mycobacterial infection, however, has not yet been explored. We found that GPR84 is induced in whole blood samples from tuberculosis patients and *Mycobacterium marinum (Mm*)-infected macrophage models. Using a *Mm-wasabi* infection model in mouse tails, we found that GPR84 is an important determinant of the extent of tissue damage. Furthermore, from our studies in an in vitro macrophage *Mm* infection model, it appears that GPR84 inhibits pro-inflammatory cytokines expression and increases intracellular lipid droplet (LD) accumulation, thereby promoting intracellular bacterial survival. Our findings suggest that GPR84 could be a potential therapeutic target for host-directed anti-TB therapeutics.

## 1. Introduction

Tuberculosis (TB), caused by the *Mycobacterium tuberculosis* (*Mtb*), has re-emerged as the leading cause of death from infectious diseases worldwide, posing a significant public health threat [1]. Because *Mtb* can develop resistance to all currently used drugs, and because of the current scarcity of new candidate anti-TB drugs, there is increasing interest in host-directed therapy (HDT) to target components of the host’s immune response [2,3]. Several innovative host-directed agents target the membrane receptors that are characterized by high specificity for ligands and mediate signal recognition and transduction [4,5].

The G-protein-coupled receptors (GPCRs), as transmembrane proteins, include chemokine receptors, bioactive lipid receptors, and orphan receptors and play significant roles in the progression of mycobacterial infection [6,7,8]. The expression of chemokine receptor CCR5 is upregulated in *Mtb*-infected mouse bone marrow-derived macrophages (BMDMs) and activates the kinases Lyn and ERK to promote IL-10 production [6]. Similarly, the anti-lipolytic receptor GPR109A leads to a reduction in cyclic adenosine monophosphate (cAMP), which decreases perilipin phosphorylation. This process results in the formation of a protective layer around LDs that prevents lipolysis and facilitates the retention of *Mtb* within macrophages [7]. In contrast, certain GPCRs act against mycobacterial growth. The oxysterol-sensing receptor GPR183 inhibit *Mtb* and BCG proliferation by negatively regulating type I IFN production [9]. Additionally, in a peritoneal macrophage model of BCG infection, the deficiency of GPR160 impedes ERK signaling, reducing BCG entry and enhancing resistance in mice [8]. Collectively, these studies demonstrate that GPCRs modulate host immune pathways to either facilitate or inhibit mycobacterial growth. Moreover, a screening of host-targeted small molecules identified that 32% (41/133) of compounds that limit mycobacterial growth in macrophages are GPCR modulators [10]. This highlights the potential of GPCRs as therapeutic targets for tuberculosis treatment.

GPR84 falls within the rhodopsin-like branch of the GPCR family (Class A) and is expressed in various innate immune cell types, including neutrophils, monocytes, macrophages, and microglia [11,12]. It has been reported that GPR84 contributes to inflammatory disease processes including ulcerative colitis, acute lung injury, diabetes, and atherosclerosis [13,14,15]. In a dextran sulfate sodium (DSS)-induced acute colitis mouse model, lowered protein levels of pro-inflammatory cytokines, including IL-1β, IL-6, and TNF-α in colon tissue in *Gpr84*^−/−^ mice at later stages and mitigated mucosal damage [13], were observed. Similarly, in an LPS-induced acute lung injury (ALI) model, Yin et al. observed that alveolar macrophages transitioned from a CD11b^lo^ to a CD11b^hi^ inflammatory state at later stages, whereas *Gpr84*^−/−^ alveolar macrophages (AMs) reduced the mRNA expression of inflammatory cytokines such as IL-6, TNF, and IL-12β [14], ultimately leading to milder tissue damage. These studies indicate that GPR84 exacerbates inflammation-induced tissue damage and enhances the expression of pro-inflammatory cytokines in the later stages of disease. Furthermore, GPR84 has been implicated in the immune responses to both bacterial and viral infections [16]. For instance, BMDMs pretreated with a GPR84 agonist (6-OAU) demonstrated increased phagocytic activity against *E. coli* [15]. In a zebrafish–*Shigella* model, Torraca et al. illustrated that *gpr84* remained differentially expressed during both early (4 h) and late (24 h) stages of infection, with *gpr84*-deficient zebrafish exhibiting heightened susceptibility to *Shigella* infection [17]. In the context of viral infection, GPR84 expression was upregulated in neutrophils from COVID-19 patients, as revealed by the RNA sequencing of bronchoalveolar lavage fluid [18]. Collectively, these findings underscore the complex role of GPR84 in immune responses to pathogenic infections. GPCRs are important targets for drug development, and identifying the specific GPCR targets involved in the progression of TB is crucial for drug development [10]. Through the analysis of publicly available RNA-seq datasets, we found that GPR84 is upregulated in the very early stages of mycobacteria infection (2h or 4h), suggesting that GPR84 may play a key role in the establishment of mycobacteria infection [19,20]. However, no studies have yet reported on the function of GPR84 in mycobacterium infection. Therefore, we conducted an initial exploration of the role and mechanism of GPR84 in mycobacterium infection, providing new insights into the host–pathogen interaction in mycobacterium infection.

We report that *GPR84* expression is markedly upregulated during mycobacterial infections. Utilizing *Mm* infections in both an in vivo mouse model and an in vitro macrophage model, we show an association between GPR84 and mycobacterial proliferation, infection-induced tissue damage, and the expression of pro-inflammatory cytokines. These findings suggest that GPR84 is integral to the pathophysiology of mycobacterial infection and represents a potential target for host-directed therapeutic strategies in the treatment of tuberculosis.

## 2. Materials and Methods

### 2.1. Collection of Blood Samples from TB Patients

Blood samples were obtained from 45 healthy controls (HC) and 48 TB patients (TB), through the Tuberculosis Department and the Physical Examination Center of Shanghai Public Health Clinical Center. Whole blood samples were collected in EDTA anticoagulant tubes, and RNA was extracted using Qiagen kit (QIAGEN, Dusseldorf, Germany). Furthermore, 1 μL of the RNA samples was used to determine the concentration and purity with the Nanodrop 2000, which was subsequently utilized for subsequent qRT-PCR analysis. The sample collection was approved and followed ethical guidelines by Shanghai Public Health Clinical Center Ethics Committee, Fudan University (Number: 2019-S009-02). Informed consent was obtained during the collection process.

### 2.2. Bacteria Strain

The *Mm-wasabi* and *Mm-tdTomato* were generously provided by Lalita Ramakrishnan from the University of Washington [21] and Professor Stefan H. Oehlers [22], respectively. The *Mm-wasabi* and *Mm-tdTomato* strains were cultivated in 7H9 medium supplemented with 10% OADC and 50 µg/mL hygromycin (Hyg) at a temperature of 32 °C. Upon reaching the logarithmic growth phase, indicated by an OD_600_ of 0.6 to 0.8, the bacterial cultures were subjected to sonication using a disperser. The ultrasound parameters employed included sonication for 15 s, separated by a 10 s pause, with a total treatment duration of 1 min [23].

### 2.3. Cell Culture

In this study, three distinct types of macrophages were utilized: the mouse macrophage cell line RAW264.7, the human macrophage cell line THP-1, and mouse bone marrow-derived macrophages (BMDMs). RAW264.7 cells were cultured in a DMEM medium containing 10% fetal bovine serum (FBS) and 1% penicillin/streptomycin (P/S). THP-1 cells were maintained in 1640 medium, also supplemented with 10% FBS and 1% P/S, and were differentiated with PMA for 48 h prior to experimentation. All cell cultures were incubated at 37 °C in a 5% CO_2_ atmosphere [24].

BMDMs were isolated from the tibias and femurs of 6- to 8-week-old female C57BL/6 WT and *Gpr84*^−/−^ mice. In brief, fresh bone marrow cells were extracted and cultured in the DMEM medium containing 10% FBS, 1% P/S, and 100 ng/mL M-CSF for a duration of 7 days. Following this incubation period, the mature BMDMs were harvested and subsequently seeded into various culture dishes or plates as required for the experimental procedures [25].

### 2.4. Infection of Macrophages

The bacterial inoculum for the infection of macrophages was determined according to the specific experimental design [26]. For the collection of RNA samples, macrophages were seeded 6 × 10^5^ cells/well in a 12-well plate and infected with *Mm-wasabi* at a MOI of 10. For intracellular bacterial proliferation, BMDMs were seeded 6 × 10^5^ cells/well in a 12-well plate and infected at an MOI of 1. Following infection, BMDMs were treated with 200 µg/mL of gentamicin at 4 hpi to eliminate extracellular bacteria. BMDMs were then lysed with 0.1% Triton X-100 at 6 hpi, 24 hpi, and 48 hpi, respectively. The released bacteria were plated onto 7H10 agar plates and were counted for 2 weeks.

### 2.5. qRT-PCR Detection of GPR84 mRNA Expression

Total RNA was isolated using TRIzol reagent [24], and RNA concentration was measured using a Nanodrop spectrophotometer. The RNA was then stored at −80 °C. Reverse transcription was performed using a reverse transcription kit according to the manufacturer’s instructions to convert RNA into cDNA. Quantitative RT-PCR was then performed using SYBR Green Supermix on the CFX96 Real-Time System (BioRad, Hercules, CA, USA). The sequences of the primers used are shown in Table 1.

The relative expression of the target gene was calculated using the comparative threshold cycle (CT) method, specifically the 2^−ΔΔCT^ method. The formula is as follows [24]:

ΔΔCT = Experimental sample (CT_target gene_ − CT_β-actin_) − Control sample (CT_target gene_ − CT_β-actin_).

### 2.6. Animals

Female wild-type (WT) mice were purchased from Shanghai Jihui Co., Ltd. (Shanghai, China). The *Gpr84* knockout (*Gpr84*^−/−^) mice were kindly provided by Professor Hua Ren from East China Normal University [27]. Mice were maintained under standard specific pathogen-free (SPF) conditions, with a temperature of 24 °C, humidity levels ranging from 45% to 55%, and a 12 h light/dark cycle, allowing for ad libitum access to food and water. All female mice utilized in the experiments were aged between 6 and 8 weeks and were randomly selected and maintained into different mouse cages. Three experiment members joined the mouse tail vein infection process, and Member A randomly selected a mouse, Member B numbered the group “WT group” and “*Gpr84*^−/−^ group”, and Member C conducted tail veins injection. The animal infection experiments were approved by Shanghai Public Health Clinical Center Laboratory Animal Welfare & Ethics Committee, Fudan University (Number: 2021-A051-01).

### 2.7. Mouse Infected with Mm-Wasabi, Histopathological Analysis, Nile-Red Staining, and Acid-Fast Staining

#### 2.7.1. Mouse Infected with Mm-Wasabi

The tail veins of 6 to 8-week-old mice were injected with 4 × 10^7^ colony-forming units (CFU) of *Mm-wasabi*, with phosphate-buffered saline (PBS) as a negative control. Tissue damage in the tails of the mice was evaluated visually every other day over a total of 14 days, after which the mice were euthanized and the tails were harvested and examined for tissue pathology. The total area of tail ulcers (mm^2^) was calculated as length (mm) × width (mm) [24].

#### 2.7.2. Histopathological Analysis

The collected mouse tail samples were fixed in 4% paraformaldehyde and embedded in paraffin. Then, 5 µm thick sections were stained with hematoxylin and eosin (H&E) and examined by light microscopy [24]. The *Mm* burdens in the mouse tail were calculated by acid-fast staining according to standard Ziehl–Neelsen method, and they were visualized by light microscopy [28].

#### 2.7.3. Nile Red Staining

The collected mouse tail samples were fixed in 4% paraformaldehyde and subsequently embedded in paraffin. Tissue sections were deparaffinized in xylene for 10 min, repeated three times, and then rehydrated using a gradient of ethanol (100%, 85%, and 75%) with each concentration maintained for 5 min, followed by a wash with distilled water. The sections were then incubated with Nile Red dye (1 µg/mL) (AbMole, M5118) (37 °C, 30 min), and the nuclei were stained with DAPI (RT, 10 min) [29,30]. Fluorescent images were acquired using a Nikon inverted fluorescence microscope.

#### 2.7.4. Immunofluorescence Assay

Mouse tail sections, deparaffinized and rehydrated as above, were treated with an EDTA antigen retrieval solution for 30 min at 100 °C and at a pH of 9.0. Following this, the sections were blocked with 3% BSA, incubated with primary antibodies (5 µg/mL, overnight), and incubated with secondary antibodies. Finally, DAPI was applied for nuclei stained [31]. Fluorescent images were observed and captured using a Nikon inverted fluorescence microscope.

### 2.8. Immunofluorescence Microscopy Imaging

For immunofluorescence assays, cells were seeded into glass-bottom dish (Thermo Scientific™, Waltham, MA, USA, 150682). After completion of the infection assay, the dishes were fixed with 4% paraformaldehyde, permeabilized with 0.2% Triton X-100, and then blocked with 5% goat serum in PBS. The dishes were then incubated with BODIPY and counterstained with DAPI. Images were visualized using a Leica SP8 confocal microscope (Laica, Wetzlar, Germany).

### 2.9. Flow Cytometry

*Mm-tdTomato* (MOI = 10) were added to BMDM for 4 h, and non-infected bacteria were eliminated with 200 µg/mL of gentamicin for 2 h. The BMDM were then treated with 1 mg/mL BODIPY 493/503 (Invitrogen D3922, Carlsbad, CA, USA) (RT, 15 min) and stained with a dead/live dye (FVD eFluor 780, Invitrogen 65-0865) (1:1000, RT, 10 min). Subsequently, BMDM were washed and fixed with 3% paraformaldehyde (RT, 30 min), resuspended in 100 µL PBS, and further analyzed for LDs accumulation using the BD LSRFortessa flow cytometer (BD, Franklin Lakes, NJ, USA) [32].

### 2.10. RNA Sequencing and Transcriptome Analysis

WT-BMDM and *Gpr84*^−/−^ BMDM infected with *Mm-wasabi* were collected at 4 hpi and 36 hpi, and RNA extracted using TRIzol reagent. Transcriptome sequencing and analysis were performed by Shanghai OE Biotechnology Co., Ltd. (Shanghai, China). In brief, libraries were sequenced on the Illumina Novaseq 6000 platform (Illumina, San Diego, CA, USA), and the principal component analysis (PCA) of gene counts was conducted using R (v3.2.0) to assess biological replication across samples.

HISAT2 software (v2.1.0) was used for alignment to the reference genome (NCBI_GRCm39) [33]. Differentially expressed genes (DEGs) were identified using DESeq2 (v1.22.2) with a q-value < 0.05 and fold change > 2 or <0.5 [34]. Gene Ontology (GO) [35] and Kyoto Encyclopedia of Genes and Genomes (KEGG) [36] pathway analyses were performed using hypergeometric distributions to identify significantly enriched functional categories. Gene Set Enrichment Analysis (GSEA) [37] was conducted using predefined gene sets, and genes were ranked based on their differential expression between sample groups.

### 2.11. Data Analysis

GraphPad Prism 9 software was used to analyze the data, presented as Mean ± SEM. Image J (v1.8.0) software was used to analyze the fluorescence images. Student’s *t*-test (two-tailed), one-way ANOVA with Tukey’s multiple comparisons test, and two-way ANOVA with multiple comparisons were performed to test the statistical significance. *p* < 0.05 was considered significant.

## 3. Results

### 3.1. Mycobacterial Infection Induces Upregulation of GPR84 Expression

**Figure 1:**
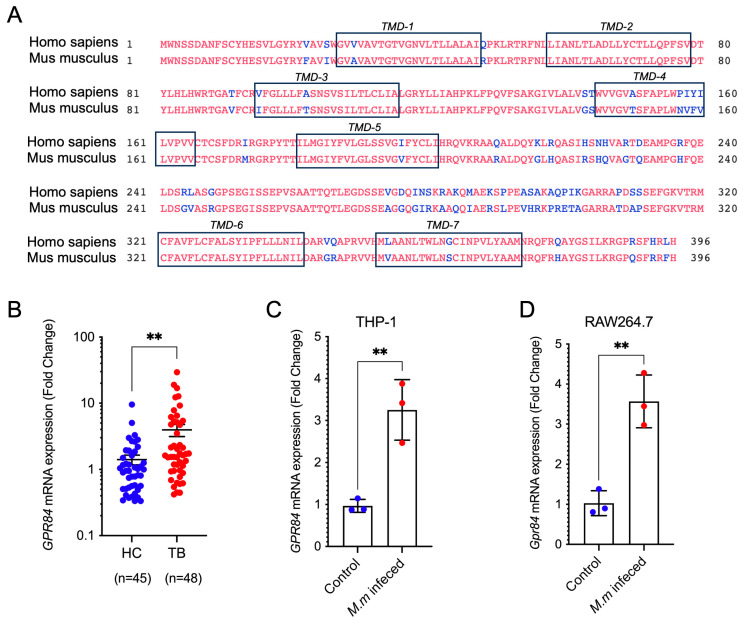
Mycobacterial infection induces a significant increase in *GPR84* mRNA expression. (**A**): comparison of mouse GPR84 homologous proteins with human GPR84. The differential amino acid is marked in blue, and the seven transmembrane sequences are marked in the black box. (**B**): *GPR84* mRNA expression in the blood of 48 tuberculosis patients (TB, n = 48) and 45 healthy controls (HC, n = 45). (**C**): *GPR84* mRNA expression in PMA-differentiated THP- 1 macrophages infected with *Mm-wasabi* (MOI = 10) compared to non-infected controls at 6 hpi. Total RNA was isolated and subjected to RT-PCR. (**D**): *Gpr84* mRNA expression in RAW264.7 macrophages infected with *Mm-wasabi* (MOI = 10) compared to non-infected controls at 6hpi. Total RNA was isolated and subjected to RT-PCR. Values were normalized to *β-actin*. Data were analyzed with GraphPad Prism 9 software and are presented as mean ± SEM. Data are representative of three independent experiments. Statistical analysis was conducted using Student’s *t*-test for (**B**–**D**). **: *p* < 0.01.

To investigate the role of GPR84 in TB, we compared the amino acid sequence of the GPR84 homolog in mice with that of human GPR84. The results showed that the amino acid sequences of the seven transmembrane regions are highly conserved among these species (Figure 1A). Next, we explored GPR transcript changes during *Mtb* infections with publicly available data, and GPR84 is significantly upregulated among RAW264.7 infected with different MOIs [19] and H37Rv-infected AM [20] at early time points of infection (4 h or 2 h). Subsequently, we assessed the expression of GPR84 mRNA in whole blood samples obtained from patients diagnosed with tuberculosis. The results indicated a significant upregulation of *GPR84* mRNA in the blood of these patients (Figure 1B), suggesting the potential involvement of GPR84 in the progression of mycobacterial infection. Following this, we validated these results in vitro using mice and human macrophage cell lines infected with *Mm-wasabi*, which similarly demonstrated a significant increase in *Gpr84* mRNA expression post-infection (Figure 1C,D). These findings corroborate that the upregulation of GPR84 is positively correlated with mycobacterial infection, implying that GPR84 may play a critical role in the mycobacterial infection process within the host.

### 3.2. GPR84 Aggravates Mycobacterium-Induced Tissue Damage

**Figure 2:**
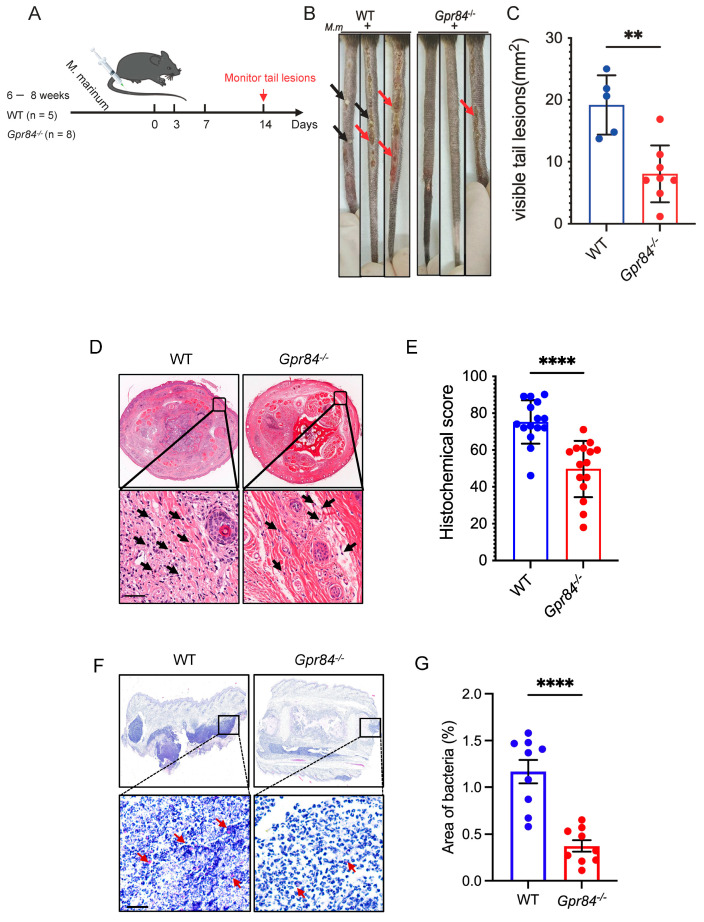
Tail pathology in mice following *Mm-wasabi* infection. (**A**): Schematic of the experimental procedure for tail vein infection with *Mm-wasabi* in mice. *Gpr84*^−/−^ (n = 8) and WT (n = 5) mice were infected via tail vein injection with 4 × 10^7^ CFU of *Mm-wasabi*. Tail lesions were monitored and photographed every other day, with comprehensive tail images of the entire tail captured at 14 dpi. (**B**): Three representative images of tails of WT and *Gpr84*^−/−^ mice at 14 dpi. Black arrows indicate nodules and red arrows indicate denote ulcerations. (**C**): Quantification of area of tail lesions, calculated by length (mm) × width (mm), as shown in panel (**B**). Each data point represents an individual mouse tail. (**D**): Representative histopathological images of H & E stained tail tissue from WT and *Gpr84*^−/−^ mice at 14 dpi. The analysis was performed using five mice per group, with three sections examined per mice. Black arrows indicate infiltrated immune cells. Scale bar: 100 µm. (**E**): Quantification of immune cell infiltration in tail tissue sections. The analysis includes five mice per group, with three tissue sections examined per mice. (**F**): Representative images of acid-fast staining of *Mm-wasabi*. The analysis was performed using three mice per group, with three sections examined per mice. Red arrows indicate bacteria. Scale bar, 100 μm. (**G**): Quantification of area of red-marked bacteria by ImageJ (v1.8.0) software. Data were analyzed with GraphPad Prism 9 software and are presented as mean ± SEM. Statistical analysis was performed using Student’s *t*-test for (**C**,**E**,**G**). ** *p* < 0.01, **** *p* < 0.0001.

We subsequently employed an in vivo mouse tail vein infection model to evaluate the effects of *Gpr84*^−/−^ mice on tissue pathology following *Mm-wasabi* injection into the tail vein (Figure 2A) [24]. Ulceration was observed on the tails of all mice at 14 dpi; however, overall, tail lesions on *Gpr84*^−/−^ mice were significantly reduced in comparison to the tail lesions of WT mice (Figure 2B,C). WT mice displayed elongated and continuous lesions with swelling, extensive skin ulceration, and white nodules. In contrast, *Gpr84*^−/−^ mice exhibited only mild skin damage characterized by sparsely distributed lesions and minimal ulceration, resulting in significantly smaller areas of ulceration (Figure 2B,C). The histological analysis of tail tissue sections, along with counts of immune cells, indicated a decreased level of immune cell infiltration in *Gpr84*^−/−^ mice (Figure 2D,E). Moreover, *Gpr84*^−/−^ mice infected with *Mm-wasabi* exhibited lower *Mm* loads in their tails examined by acid-fast staining (Figure 2F,G). These results suggest that GPR84 plays an important role in mediating tissue damage and promoting infection.

### 3.3. GPR84 Promotes Intracellular Proliferation of Mm-Wasabi and LD Accumulation

**Figure 3:**
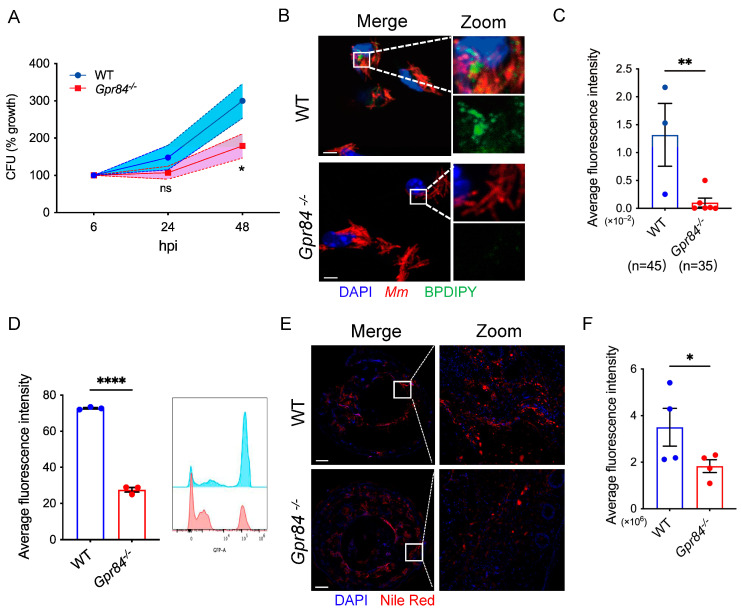
GPR84 deficiency reduces LDs accumulation in macrophages. (**A**): Colony-forming unit (CFU) counts of WT and *Gpr84*^−/−^ BMDM infected *Mm-wasabi* at an MOI of 1, lysed at 6 hpi, 24 hpi, and 48 hpi; CFU were counted on plates. Data are representative of three independent experiments. (**B**): Confocal microscopy images of lipid content in WT and *Gpr84*^−/−^ BMDMs at 6 hpi with *Mm-tdTomato* (MOI = 10). *Mm-tdTomato* is shown in red, BODIPY-stained lipids in green, and DAPI-stained nuclei in blue. The images were representative of three independent experiments. Scale Bar: 5 µm. (**C**): Quantification of LDs in macrophages based on green BODIPY fluorescence intensity, analyzed by ImageJ (v1.8.0) software. (**D**): Flow cytometry analysis of LDs in macrophages 6 hpi with *Mm-tdTomato* (MOI = 10). The left panel shows the percentage of BODIPY-positive macrophages, and the right panel shows BODIPY-positive fluorescence intensity. (**E**): Representative multi-color immunofluorescence staining of tail tissue sections from mice at 14 dpi. LDs were stained with Nile Red (red) and cell nuclei with DAPI (blue). The images were representative of three independent experiments. Scale Bar: 100 µm. (**F**): Quantification of red fluorescence intensity of Nile Red-stained LDs by ImageJ (v1.8.0) software. Data were analyzed with GraphPad Prism 9 software and are presented as mean ± SEM. Statistical significance was determined by two-way ANOVA for (**A**) and Student’s *t*-test for (**C**,**D**,**F**). * *p* < 0.05, ** *p* < 0.01, **** *p* < 0.0001.

BMDMs from *Gpr84*^−/−^ and WT mice were infected with *Mm-tdTomato* at an MOI of 1, and intracellular bacterial proliferation was assessed at 6, 24, and 48 hpi. *Gpr84*^−/−^ BMDMs demonstrated significantly lower bacterial loads in comparison to WT BMDMs (Figure 3A). Because LD accumulation can influence mycobacterial growth within macrophages [38], we hypothesized that *GPR84* deficiency might also affect LD accumulation in macrophages. Using BODIPY staining to visualize LD accumulation in BMDMs, we found that following *Mm-tdTomato* infection induced the formation of LDs, which co-localized with *Mm-tdTomato*. Compared to WT BMDM, *Gpr84*^−/−^ BMDMs exhibited less co-localization of bacteria with LDs (Figure 3B) and a reduced BODIPY fluorescence intensity, indicating fewer LDs (Figure 3C). Furthermore, flow cytometry analysis confirmed a significant reduction in the number of BODIPY+ macrophages within the *Gpr84*^−/−^ group (Figure 3D). Similarly, the histological examination of sections from the tail lesions revealed significantly less LD accumulation in the lesions of *Gpr84*^−/−^ mice (Figure 3E,F) compared to WT mice.

### 3.4. Gpr84^−/−^ BMDMs Promotes the Expression of Pro-Inflammatory Cytokine

**Figure 4:**
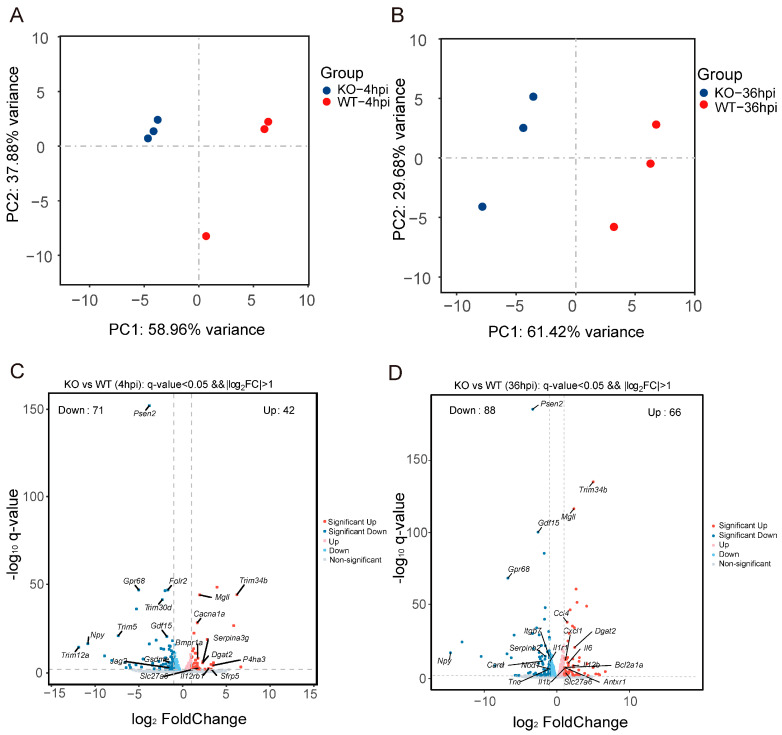

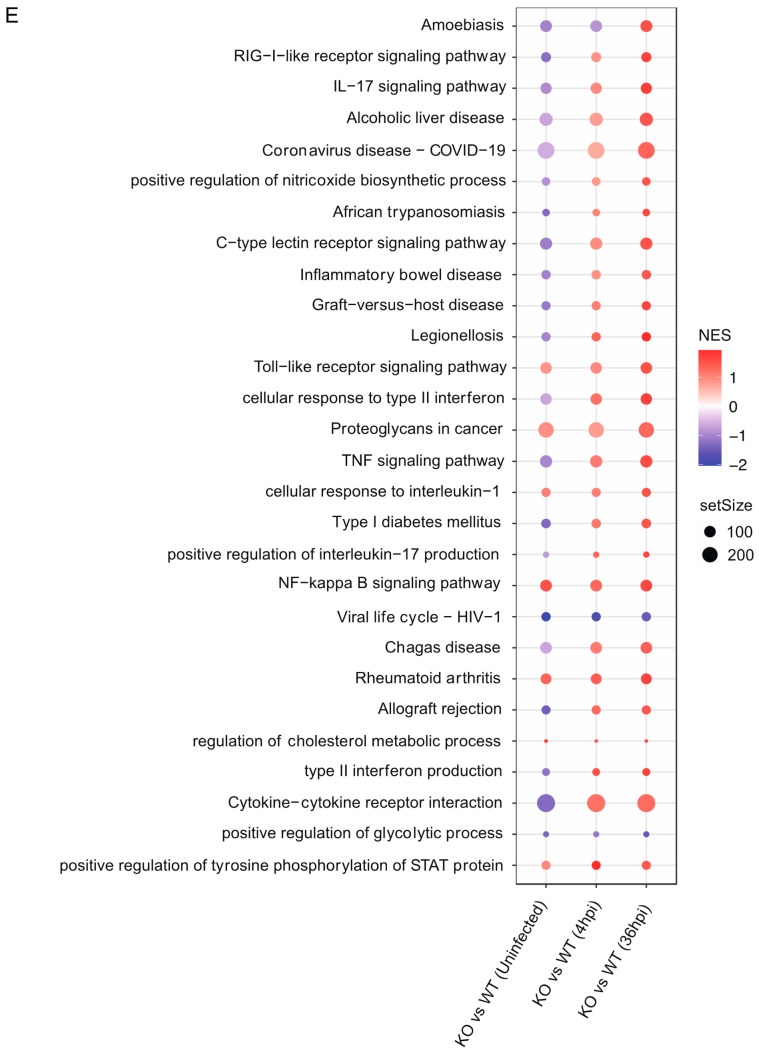
Results of RNA-seq analysis, differentially expressed genes and pathways. (**A**,**B**): Multidimensional scaling between *Gpr84*^−/−^ BMDM and WT BMDM at 4 hpi (**A**) and 36 hpi (**B**). Each group has three biological replicates. (**C**,**D**): Volcano plot of differentially expressed genes (DEGs). Genes with significant expression differences (*p* < 0.05) are listed, while red dots and blue dots are up- or downregulated genes in *Gpr84*^−/−^ BMDM, respectively, compared to WT BMDM at 4 hpi (**C**) and 36 hpi (**D**). The *x* axis represents log_2_ of fold change and the *y* axis represents the log_10_ of *p* values. (**E**): KEGG pathway enrichment analysis of significant DEGs in uninfected and *Mm*-infected BMDM at 4 hpi and 36 hpi. KEGG pathway enrichment analysis of significant DEGs in datasets from uninfected BMDM, *Mm*-infected BMDM at 4 hpi and 36 hpi. Set size represents the number of DEGs. Normalized enrichment score (NES) represents the gene enriched degree, and higher |NES| represent more up- or downregulated genes in a given pathway.

**Figure 5:**
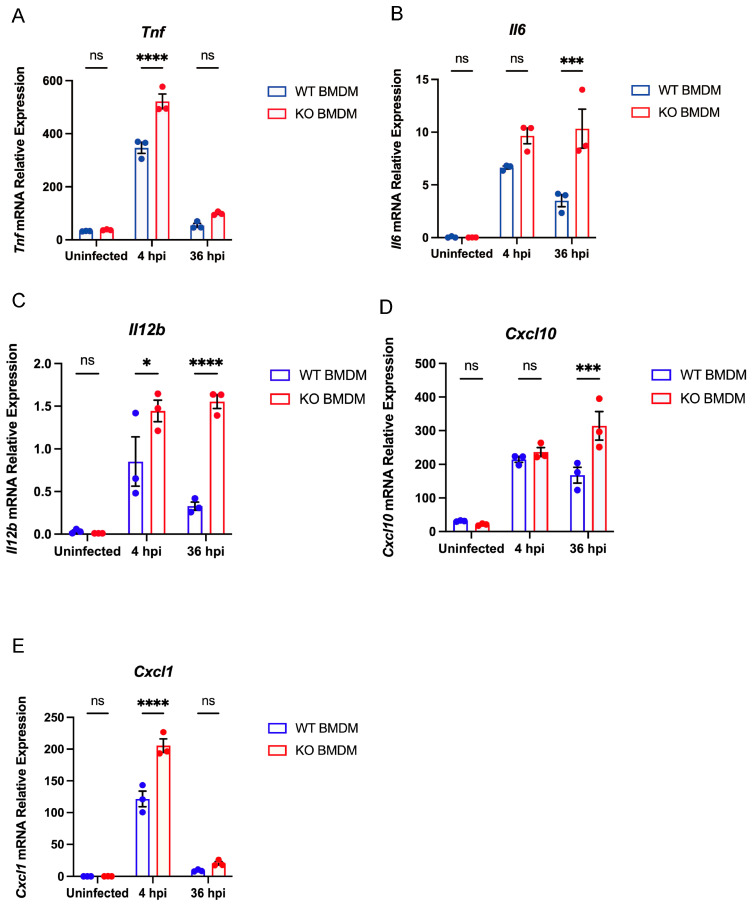
*Gpr84*^−/−^ BMDMs promotes the expression of pro-inflammatory cytokines. (**A**–**E**): *Tnf*, *Il6, Il12b*, *Cxcl10,* and *Cxcl1* mRNA expression. Based on the RNA-seq analysis results, the expression of *Tnf* (**A**), *Il6* (**B**), *Il12b* (**C**), *Cxcl10* (**D**), and *Cxcl1* (**E**) mRNA was quantitated in WT and *Gpr84*^−/−^ BMDM. Data were analyzed with GraphPad Prism 9 software and are presented as mean ± SEM. Statistical analysis was performed using two-way ANOVA for (**A**–**E**). * *p* < 0.05, *** *p* < 0.001, **** *p* < 0.0001, ns, not significant.

To further explore the factors contributing to the limited proliferation of mycobacterium in *Gpr84*^−/−^ BMDMs, we conducted a systematic analysis of differentially expressed genes (DEGs) in *Mm*-infected WT and *Gpr84*^−/−^ BMDMs using RNA-seq technology. WT and *Gpr84*^−/−^ BMDM samples were effectively differentiated through multidimensional scaling (Figure 4A,B). Compared to WT BMDMs, 25 genes were significantly upregulated in *Gpr84*^−/−^ BMDMs at both 4 hpi and 36 hpi. Among these, *Adamts1* [39], *Bex1* [40], *Col5a3* [41], *Mid1* [42], and *Tie1* [43] have previously shown to be highly expressed during inflammatory responses and exhibit pro-inflammatory characteristics, which are closely associated with an enhanced host bactericidal function. Additionally, the genes *Mgll* [44], *Dgat2* [45], *Slc27a6* [45], and *Stard10* [45] have been implicated in lipid metabolism, indicating that lipid metabolism may be disrupted in *Gpr84*^−/−^ BMDMs during mycobacterial infection. Notably, among the 66 upregulated genes identified in *Gpr84*^−/−^ BMDMs at 36 hpi, there were genes associated with the NF-kappaB signaling pathway, including *Ccl4, Cxcl1, and Bcl2a1a* [46,47,48]. Other genes related to the macrophage inflammatory response, such as *Il6* and *Il12b*, also exhibited upregulation at 36 hpi but not 4 hpi. These findings suggest that *Gpr84*^−/−^ BMDMs at 36 hpi may enhance the inflammatory response in macrophages, thereby facilitating the control of mycobacterium proliferation (Figure 2G and Figure 4D). Subsequently, we utilized the KEGG database to categorize the functions of DEGs and conducted a further analysis of the variations in downstream signaling pathways. Compared to 4 hpi, BMDMs at 36 hpi exhibited enhanced enrichment in genes related to pathways seen in diseases (such as Amoebiasis, alcoholic liver disease, and COVID-19), receptor signaling (such as RIG-I-like receptor signaling pathway and C-type lectin receptor signaling pathway), IL-17 signaling pathway, and the positive regulation of nitric oxide biosynthetic pathway (Figure 4E). Genes such as *Il12b, Il6, Cxcl10, Tnf,* and *Cxcl1* are involved in multiple pathways, suggesting that the genes upregulated in *Gpr84*^−/−^ BMDMs at 36 hpi may help to control mycobacterium proliferation. Furthermore, the gene expression analysis of cytokines associated with bactericidal activity found that the mRNA expression of pro-inflammatory cytokines *Tnf, Il6, Il12b, Cxcl10,* and *Cxcl1* were significantly upregulated compared to WT BMDMs at 4 hpi (Figure 5A–E).

## 4. Discussion

In this study, we found that GPR84 plays a harmful role for hosts in mycobacterial infections. In both BMDM and mouse tail models, *Gpr84* deficiency was associated with the reduced accumulation of the intra-cellular LDs that facilitate bacterial survival within host cells. And GPR84 deficiency is beneficial to increase the expression of pro-inflammatory cytokines during mycobacterium infection.

Previous research has demonstrated that the deficiency of GPR84 mitigates host inflammatory responses [13,49,50,51]. For example, in a mouse model of ALI, *Gpr84*^−/−^ mice significantly reduced pulmonary inflammation by decreasing neutrophil recruitment and the production of reactive oxygen species (ROS) [13]. Additionally, in a DSS-induced colitis model, *Gpr84*^−/−^ mice reduced the infiltration of inflammatory cells and damage to the colonic mucosa [51]. These results are similar to our observation of decreased inflammatory cell infiltration and tissue damage in *Mm-wasabi* infected tails of *Gpr84*^−/−^ mice (Figure 2) [13,51].

Our RNA-seq results indicate that in *Gpr84*^−/−^ BMDMs following *Mm-wasabi* infection, the expression of pro-inflammatory cytokines such as *Tnf*, *Il12b*, *Il6*, *Cxcl10,* and *Cxcl1* is significantly elevated (Figure 5A–E). It is possible that the increased cytokines production enhances the ability of *Gpr84*^−/−^ BMDMs to control bacterial proliferation (Figure 3A). However, this observation appears to contradict the previous notion that GPR84 promotes the M1 polarization of macrophages and increases the expression of pro-inflammatory cytokines, including TNF-α and IL-6 [13,14]. We propose several possible explanations for this discrepancy. First, the mechanisms by which GPR84 functions following infection may differ depending on the diseases being studied. In acute lung injury, acute colitis, or LPS-induced acute inflammation, GPR84 acts as a pro-inflammatory receptor that increases the release of late-stage pro-inflammatory cytokines, exacerbating pathological damage [13,14,51]. However, in chronic diseases such as osteoarthritis, non-alcoholic steatohepatitis, and *Brucella abortus* infection, GPR84 plays a role in modulating inflammation by reducing the expression of pro-inflammatory factors (such as TNF-α), thereby alleviating disease progression or promoting infection [16,52,53]. Consistent with the findings on the aforementioned *Brucella abortus* infection [16], we also observed that GPR84 negatively regulates the inflammatory response to promote infection. Therefore, our results further emphasize the importance of disease models when studying GPR84’s function, as GPR84 may play distinctly opposite roles in different disease models. Additionally, the timing of the expression of inflammatory cytokines may differ depending on the disease models. In acute inflammatory models, the role of GPR84 in promoting pro-inflammatory functions typically manifests at later stages, where cytokine expression may indicate a secondary response in the progression of the disease [13,51]. We observed that at an early time point post infection, *Gpr84*^−/−^ mice showed an increased expression of pro-inflammatory cytokines, suggesting that GPR84 may inhibit their expression in the early stages of infection (Figure 5A–E). Although variations in cytokine expression are noted at different time points, GPR84 consistently facilitates disease progression (Figure 2B) [13,51]. Furthermore, differences in cellular models may significantly influence the role of GPR84 in the production of pro-inflammatory cytokines. For example, GPR84-mediated signaling in microglia promotes cell migration through the Gi/o pathway, but does not elicit a pro-inflammatory response [54]. Consequently, while it appears that GPR84 plays an important role in the immune response to infections, its functions are complex and may vary depending on the pathogen [15,16], the disease model [13,53], the time of the response, and the specific cell type examined [51,54].

One way by which mycobacteria appear to evade the host immune response is by promoting the accumulation of LDs within macrophages [55,56]. In macrophages infected with *Mtb*, a reduction in the accumulation of LDs restores the macrophage antibacterial capacity, leading to a reduction in the intracellular burden of *Mtb* [56,57,58]. In our study, we observed that *Gpr84*^−/−^ mice decreased LD accumulation in both BMDM and mouse tails with lower *Mm* loads (Figure 3). Similarly, previous studies have reported that the overexpression of GPR84 in RAW264.7 can promote LD formation [59]. Further analysis of our RNA-seq results revealed an upregulation of lipid metabolism-related genes, *Mgll* [44] and *Dgat2* [45], at both 4 hpi and 36 hpi (Figure 4C,D), suggesting that macrophages may attempt to compensate for lipid synthesis by upregulating the expression of *Mgll* and *Dgat2* to restore lipid homeostasis in infected *Gpr84*^−/−^ macrophages. This compensatory mechanism could provide an energy reserve for the antimicrobial activity of macrophages. Meanwhile, the LD accumulation reduction in macrophages prevents the differentiation of foam cells (FM), allowing macrophages to maintain their original bactericidal function and release pro-inflammatory cytokines such as TNF-α and IL-6 to reduce intracellular bacterial load [60].

In conclusion, our study demonstrates that GPR84 exacerbates the progression of mycobacterial infection. Both in vivo findings from mouse models and in vitro results from macrophage infections indicate that the *Gpr84*^−/−^ BMDM lead to an increased expression of inflammatory cytokines, accompanied by a decrease in LD accumulation, ultimately limiting the intracellular and in vivo proliferation of mycobacteria. Further exploration of the regulatory mechanisms by which GPR84 influences the host response to mycobacterial infection may offer novel avenues for the development of host-directed anti-tuberculosis therapeutics.

## 5. Limitations

Our study found that GPR84 deficiency restricts the progression of mycobacterial infection, but the delineation of the downstream signaling pathways activated by GPR84 during mycobacterial infections will require subsequent investigation. Furthermore, the ligand for GPR84 and whether mycobacterium harbors GPR84-specific pathogen-associated molecular patterns (PAMPs), like PDIM and Sulfolipids, or other virulence factors that may affect lipid metabolism pathways or macrophage polarization to promote infection remains to be determined. Moreover, using macrophage-specific Cre driver mice with the conditional knock out of GPR84 is necessary to eliminate changes in other cells, such as neutrophils and epithelial cells.

## Figures and Tables

**Table 1:** Primers used for qPCR. Related to Figure 1.

Gene Name	GeneBank (No.)	Forward and Reverse Primer Sequence (5′-3′)
Human *GPR84*	MT536737.1	F: TGAAGCCTAACTGTCCACCAG R: CCACATGATAGAGGCTGAGT
Human *β-actin*	PQ040393.1	F: TCACCATGGATGATGATATCGC R: ATAGGAATCCTTCTGACCCATGC
Mouse *Gpr84*	AF272948.1	F: CCACCGCTTTTGCAAGGATGT R: AACGGTAGCCCTCAACAGAG
Mouse *β-actin*	BC138611.1	F: GCCGGGACCTGACAGACTAC R: TGGCCA TCTCCTGCTCGAAG

## Data Availability

The raw data supporting the conclusions of this article will be made available by the authors on request.
